# Characterization of Glycosphingolipids in the Human Parathyroid and Thyroid Glands

**DOI:** 10.3390/ijms22137044

**Published:** 2021-06-30

**Authors:** Karin Säljö, Anders Thornell, Chunsheng Jin, Peter Stålberg, Olov Norlén, Susann Teneberg

**Affiliations:** 1 Department of Plastic Surgery, Institute of Clinical Sciences, Sahlgrenska Academy, University of Gothenburg, S-41345 Gothenburg, Sweden; karin.saljo@vgregion.se; 2 Region Västra Götaland, Sahlgrenska University Hospital, S-41345 Gothenburg, Sweden; anders.thornell@vgregion.se; 3 Department of Surgery, Institute of Clinical Sciences, Sahlgrenska Academy, University of Gothenburg, S-41345 Gothenburg, Sweden; 4 Department of Medical Biochemistry and Cell Biology, Institute of Biomedicine, Sahlgrenska Academy, University of Gothenburg, S-40530 Gothenburg, Sweden; chunsheng.jin@medkem.gu.se; 5 Department of Surgical Sciences, Uppsala University Hospital, Uppsala University, S-75185 Uppsala, Sweden; peter.stalberg@surgsci.uu.se (P.S.); olov.norlen@me.com (O.N.)

**Keywords:** thyroid glycosphingolipids, parathyroid glycosphingolipids, glycosphingolipid characterization, mass spectrometry, blood group antigens

## Abstract

As part of a systematic investigation of the glycosphingolipids in human tissues, acid and non-acid glycosphingolipids from human thyroid and parathyroid glands were isolated and characterized with mass spectrometry and binding of carbohydrate-recognizing ligands, with a focus on complex compounds. The glycosphingolipid patterns of the human parathyroid and thyroid glands were very similar. The major acid glycosphingolipids were sulfatide and the gangliosides GM3, GD3, GD1a, GD1b, GT1b and Neu5Ac-neolactotetraosylceramide, and the major non-acid glycosphingolipids were globotriaosylceramide and globoside. We also found neolactotetra- and neolactohexaosylceramide, the x_2_ glycosphingolipid, and complex glycosphingolipids with terminal blood group O and A determinants in both tissues. A glycosphingolipid with blood group Le^b^ determinant was identified in the thyroid gland, and the parathyroid sample had a glycosphingolipid with terminal blood group B determinant. Immunohistochemistry demonstrated the expression of blood group A antigens in both the thyroid and parathyroid glands. A weak cytoplasmatic expression of the GD1a ganglioside was present in the thyroid, while the parathyroid gland had a strong GD1a expression on the cell surface. Thus, the glycosylation of human thyroid and parathyroid glands is more complex than previously appreciated. Our findings provide a platform for further studies of alterations of cell surface glycosphingolipids in thyroid and parathyroid cancers.

## 1. Introduction

With an incidence of 1–2%, primary hyperparathyroidism is one of the most prevalent endocrine disorders [1]. It is caused by the autonomously increased secretion of parathyroid hormone from one or several parathyroid glands. Patients with primary hyperparathyroidism may suffer from fatigue, muscle weakness, depression, abdominal discomfort, kidney stones and osteoporosis [2]. The only curative treatment for primary hyperparathyroidism is surgical removal of all diseased glands. Accurate preoperative localization allows the use of focused parathyroid surgery, which is associated with decreased complication rates and shorter operating times [2]. Unfortunately, the accuracy of conventional methods, such as parathyroid scintigraphy, ultrasound, and four-dimensional computed tomography (4D-CT), to localize all the diseased glands is limited [3,4]. Moreover, normal parathyroid glands can most often not be identified with such methods. However, this may be enabled by the identification of cell surface parathyroid specific determinants, to which tracer molecules bind specifically. Glycosphingolipids are vital components of the plasma membrane, embedded in its outer part with their single carbohydrate chain facing the extracellular environment, making them ideal epitopes for developing new molecular markers. As the thyroid and parathyroid glands are located in close anatomical vicinity, discriminatory glycosphingolipids between the two would be advantageous for precise preoperative localization.

In the 1970s–1990s, the major glycosphingolipids of the human thyroid gland were characterized as glucosylceramide, galactosylceramide, lactosylceramide, galabiosylceramide, trihexosylceramide, globoside, sulfatide, and the gangliosides GM3, GD3, GD1a, GD1b, GT1b, and Neu5Ac-neolactotetraosylceramide [5,6,7,8,9]. However, a thorough characterization with the methods available today has not been done, and the glycosphingolipids of human parathyroid glands have not been characterized. The aim of the present study was thus to characterize the glycosphingolipids of human parathyroid and thyroid glands, with particular interest in minor complex compounds, to enable comparative analyses. The glycosphingolipids were characterized by mass spectrometry and by binding a battery of carbohydrate-recognizing ligands, and the tissue distribution of selected compounds was investigated by immunohistochemistry.

## 2. Results

### 2.1. Isolation of Glycosphingolipids from Human Parathyroid and Thyroid Glands

**Figure 1:**
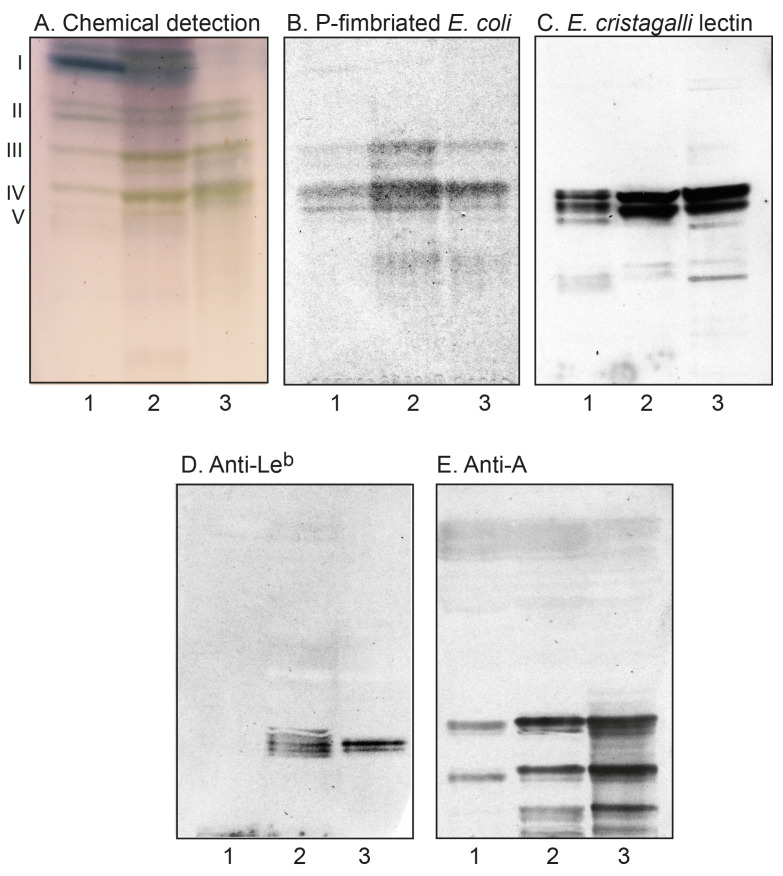
Thin-layer chromatography of the non-acid glycosphingolipids of human parathyroid and thyroid glands, and binding of carbohydrate-recognizing ligands. Thin-layer chromatogram after detection with anisaldehyde (**A**), autoradiograms obtained by binding of Galα4Gal-recognizing P-fimbriated *E. coli* (**B**), Galβ4GlcNAc-/Fucα2Galβ4GlcNAc-binding lectin from *E. cristagalli* (**C**), monoclonal antibodies directed against the blood group Le^b^ determinant (**D**), and the blood group A determinant (**E**). The lanes were: lane 1, total non-acid glycosphingolipids of human parathyroid glands, 80 μg; lane 2, total non-acid glycosphingolipids of human thyroid glands, 80 μg; lane 3, reference total non-acid glycosphingolipids of human blood group AB erythrocytes, 40 μg.The roman numbers to the left of (**A**) denote the approximate number of carbohydrate units in the bands.

**Figure 2:**
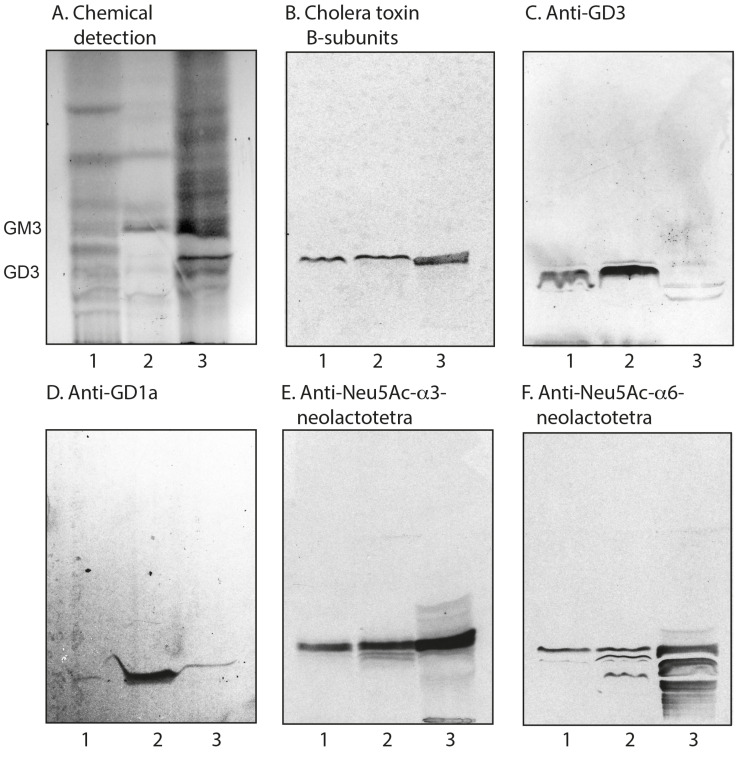
Thin-layer chromatography of the acid glycosphingolipids of human parathyroid and thyroid glands, and binding of carbohydrate-recognizing ligands. Thin-layer chromatogram after detection with anisaldehyde (**A**), autoradiograms obtained by binding of ganglioside GM1-recognizing cholera toxin B-subunits (**B**), monoclonal antibodies directed against the ganglioside GD3 (**C**), the ganglioside GD1a (**D**), the Neu5Acα3Galβ4GlcNAc sequence (**E**), and the Neu5Acα6Galβ4GlcNAc sequence (**F**). The lanes were: lane 1, total acid glycosphingolipids of human parathyroid glands, 80 μg; lane 2, total acid glycosphingolipids of human thyroid glands, 80 μg; lane 3, reference total acid glycosphingolipids of human liver cancer lung metastasis, 40 μg. The designations GM3 and GD3 to the left of (**A**) denote the migration levels of the GM3 and GD3 gangliosides, respectively.

Acid and non-acid glycosphingolipids were isolated from parathyroid glands and thyroid glands using standard methods. The amounts obtained are given in Table 1. Thin-layer chromatography with chemical detection showed that the major bands of both non-acid fractions migrated in the mono- to tetraglycosylceramide regions (Figure 1A, lanes 1 and 2). The total acid fractions both had several bands (Figure 2A, lanes 1 and 2). The slow-migrating bands were stained by resorcinol, indicating the presence of sialic acid (not shown).

The total glycosphingolipid fractions were first characterized by mass spectrometry and by binding of carbohydrate-recognizing reagents, as described below.

### 2.2. Characterization of the Non-Acid Glycosphingolipids from Human Parathyroid and Thyroid Glands

#### 2.2.1. Mass Spectrometry

Aliquots of the total non-acid glycosphingolipid fractions from human thyroid and parathyroid glands were hydrolyzed with *Rhodococcus* endoglycoceramidase II, and the free oligosaccharides thereby obtained were analyzed by liquid chromatography electrospray ionization mass spectrometry (LC-ESI/MS). LC-ESI/MS of oligosaccharides using graphitized carbon columns gives resolution of isomeric oligosaccharides, and the carbohydrate sequence can be deduced from series of C-type ions obtained by MS^2^. Furthermore, diagnostic cross-ring ^0,2^A-type fragment ions are present in the MS^2^ spectra of oligosaccharides with a Hex or HexNAc substituted at C-4, and thus allow identification of linkage positions [10,11].

**Figure 3:**
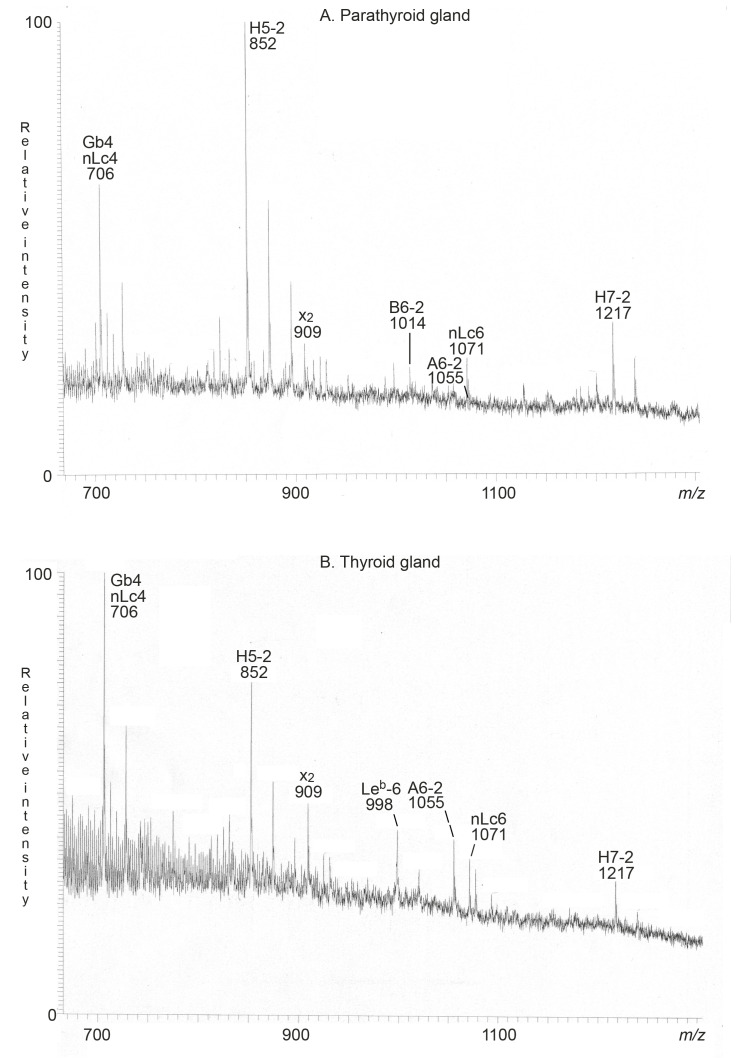
LC-ESI/MS of the oligosaccharides obtained from the total non-acid glycosphingolipid fractions from human parathyroid and thyroid glands by hydrolysis with endoglycoceramidase II from *Rhodococcus* spp. (**A**) Molecular ion profile from LC-ESI/MS of the oligosaccharides from human parathyroid glands. (**B**) Molecular ion profile from LC-ESI/MS of the oligosaccharides from human thyroid glands. The identification of oligosaccharides was based on their retention times, determined molecular masses, and subsequent MS^2^ sequencing. The oligosaccharides identified in the chromatograms were: Gb4, GalNAcβ3Galα4Galβ4Glc; nLc4, Galβ4GlcNAcβ3Galβ4Glc; H5-2, Fucα2Galβ4GlcNAcβ3Galβ4Glc; x_2_, GalNAcβ3Galβ4GlcNAcβ3Galβ4Glc; nLc6, Galβ4GlcNAcβ3Galβ4GlcNAcβ3Galβ4Glc; H7-2, Fucα2Galβ4GlcNAcβ3Galβ4GlcNAcβ3Galβ4Glc; Le^b^-6, Fucα2Galβ3(Fucα4)GlcNAcβ3Galβ4Glc; A6-2, GalNAcα3(Fucα2)Galβ4GlcNAcβ3Galβ4Glc.

**Figure 4:**
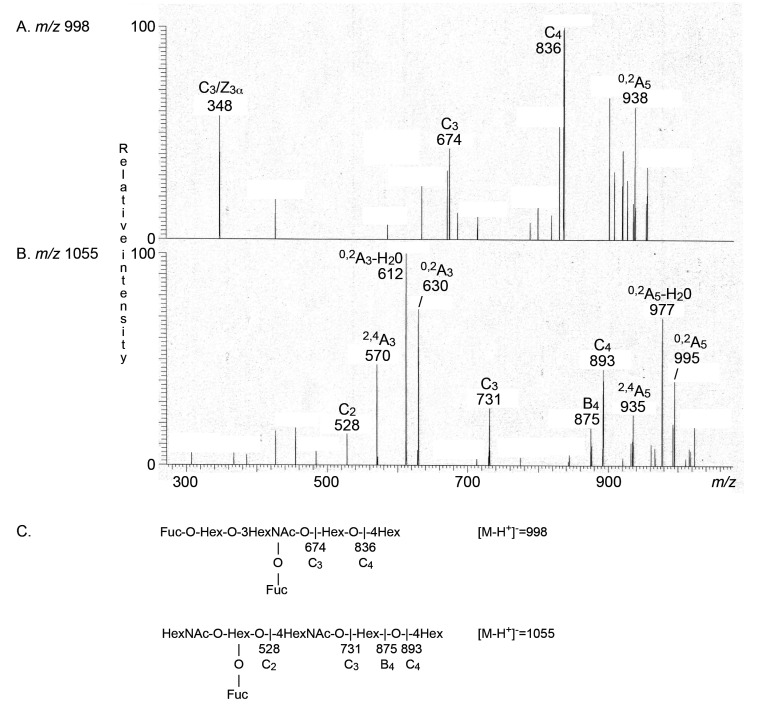
LC-ESI/MS of the oligosaccharides obtained from the total non-acid glycosphingolipid fraction from human thyroid gland by hydrolysis with endoglycoceramidase II from *Rhodococcus* spp. (**A**) MS^2^ of the ion at *m*/*z* 998. The MS^2^ spectrum had a prominent fragment ion at *m*/*z* 348. This ion is diagnostic for an internal 3-linked GlcNAc substituted with a Fuc at C-4 [11], and is a double glycosidic cleavage of the 3-linked branch at C_3_ and Z_3α_. C-type fragment ions were present at *m*/*z* 674 (C_3_) and *m*/*z* 836 (C_4_), and taken together this indicated a Le^b^ hexasaccharide (Fucα2Galβ3(Fucα4)GlcNAcβ3Galβ4Glc). (**B**) MS^2^ of the ion at *m*/*z* 1055 gave a series of C-type fragment ions (C_2_ at *m*/*z* 528, C_3_ at *m*/*z* 731, and C_4_ at *m*/*z* 893), demonstrating a HexNAc-(Fuc-)Hex-HexNAc-Hex-Hex sequence. A type 2 core chain was identified by the ^0,2^A_3_ ion at *m*/*z* 630, and the ^0,2^A_3_-H_2_O ion at *m*/*z* 612. Taken together, these spectral features identified a blood group A type 2 hexasaccharide (GalNAcα3(Fucα2)Galβ4GlcNAcβ3Galβ4Glc). (**C**) Interpretation formulas.

The major non-acid glycosphingolipids of the human thyroid gland were, in the 1970s, characterized as glucosylceramide, lactosylceramide, globotriaosylceramide, and globotetraosylceramide [5]. Here, we searched for complex compounds, focusing on tetrasaccharides and larger oligosaccharides. The molecular ion profiles of these oligosaccharides, obtained from the non-acid glycosphingolipids of the human parathyroid and thyroid glands, were very similar (Figure 3A,B), and MS^2^ sequencing of the molecular ions identified globo and neolacto tetrasaccharides (*m*/*z* 706), H type 2 pentasaccharide (*m*/*z* 852), the x_2_ pentasaccharide (*m*/*z* 909), neolacto hexasaccharide (*m*/*z* 1071), and the blood groups A type 2 hexasaccharide (*m*/*z* 1055) and H type 2 heptasaccharide (*m*/*z* 1217) in both the parathyroid gland and the thyroid gland. MS^2^ of the ion at *m*/*z* 1055 from the thyroid sample, identifying the blood group A type 2 hexasaccharide, is shown in Figure 4B.

There was also a molecular ion at *m*/*z* 1014 in the molecular ion profile from the parathyroid gland (Figure 3A), and the molecular ion profile from the thyroid gland (Figure 3B) had a molecular ion at *m*/*z* 998. MS^2^ of the ion at *m*/*z* 998 identified the Le^b^ hexasaccharide (Figure 4A). The MS^2^ spectrum obtained from the ion at *m*/*z* 1014 was very weak, and did not allow a safe interpretation.

**Figure 5:**
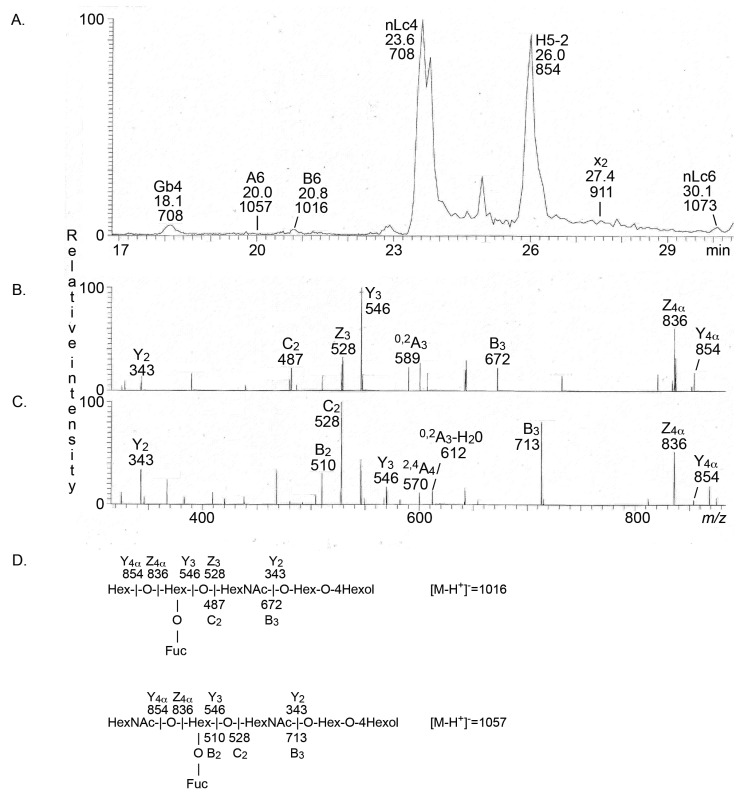
LC-ESI/MS of the reduced oligosaccharides obtained from the total non-acid glycosphingolipid fraction from human parathyroid gland by hydrolysis with endoglycoceramidase II from *Rhodococcus* spp. (**A**) Base peak chromatogram from LC-ESI/MS of the reduced oligosaccharides obtained from human parathyroid gland. (**B**) MS^2^ of the ion at *m*/*z* 1016 at retention time 20.8 min. The MS^2^ spectrum had a C_2_ fragment ion at *m*/*z* 487, and a B_3_ ion at *m*/*z* 672, demonstrating a terminal Hex-(Fuc-)Hex-HexNAc sequence. A type 2 core chain was identified by the ^0,2^A_3_ ion at *m*/*z* 589. A terminal Hex was shown by the Y^4α^ ion at *m*/*z* 854, and the Z^4α^ ion at *m*/*z* 836, and there was also a Y_3_ ion at *m*/*z* 546, and a Y_2_ ion at *m*/*z* 343. Taken together, this demonstrated a blood group B type 2 hexasaccharide (Galα3(Fucα2)Galβ4GlcNAcβ3Galβ4Glc). (**C**) MS^2^ of the ion at *m*/*z* 1057 at retention time 20.0 min. The MS^2^ spectrum had a B_2_ fragment ion at *m*/*z* 510, a C_2_ fragment ion at *m*/*z* 528, and a B_3_ ion at *m*/*z* 713, demonstrating a terminal HexNAc-(Fuc-)Hex-HexNAc sequence. The ^0,2^A_3_ fragment ion at *m*/*z* 713 showed that the internal HexNAc was substituted at C-4, i.e., a type 2 chain. There was also a Y_3_ ion at *m*/*z* 546, a Y_2_ ion at *m*/*z* 343, a Z^4α^ ion at *m*/*z* 836, and a Y^4α^ ion at *m*/*z* 854. Taken together, this demonstrated a blood group A type 2 hexasaccharide (GalNAcα3(Fucα2)Galβ4GlcNAcβ3Galβ4Glc). (**D**) Interpretation formulas showing the deduced oligosaccharide sequence. The oligosaccharides identified in the chromatograms were: Gb4, GalNAcβ3Galα4Galβ4Glc; A6-2, GalNacα3(Fucα2)Galβ4GlcNAcβ3GalβGlc; B6-2, Galα3(Fucα2)Galβ4GlcNAcβ3GalβGlc; nLc4, Galβ4GlcNAcβ3Galβ4Glc; H5-2, Fucα2Galβ4GlcNAcβ3Galβ4Glc; x_2_, GalNAcβ3Galβ4GlcNAcβ3Galβ4Glc; nLc6, Galβ4GlcNAcβ3Galβ4GlcNAcβ3Galβ4Glc.

Therefore, the sample from the parathyroid gland was reduced in an attempt to increase the sensitivity. MS^2^ of the ion at *m*/*z* 1016 (reduced *m*/*z* 1014) identified the blood group B type 2 hexasaccharide (Figure 5B), whereas the blood group A type 2 hexasaccharide was characterized by MS^2^ of the ion at *m*/*z* 1057 (reduced *m*/*z* 1055) (Figure 5C).

**Figure 6:**
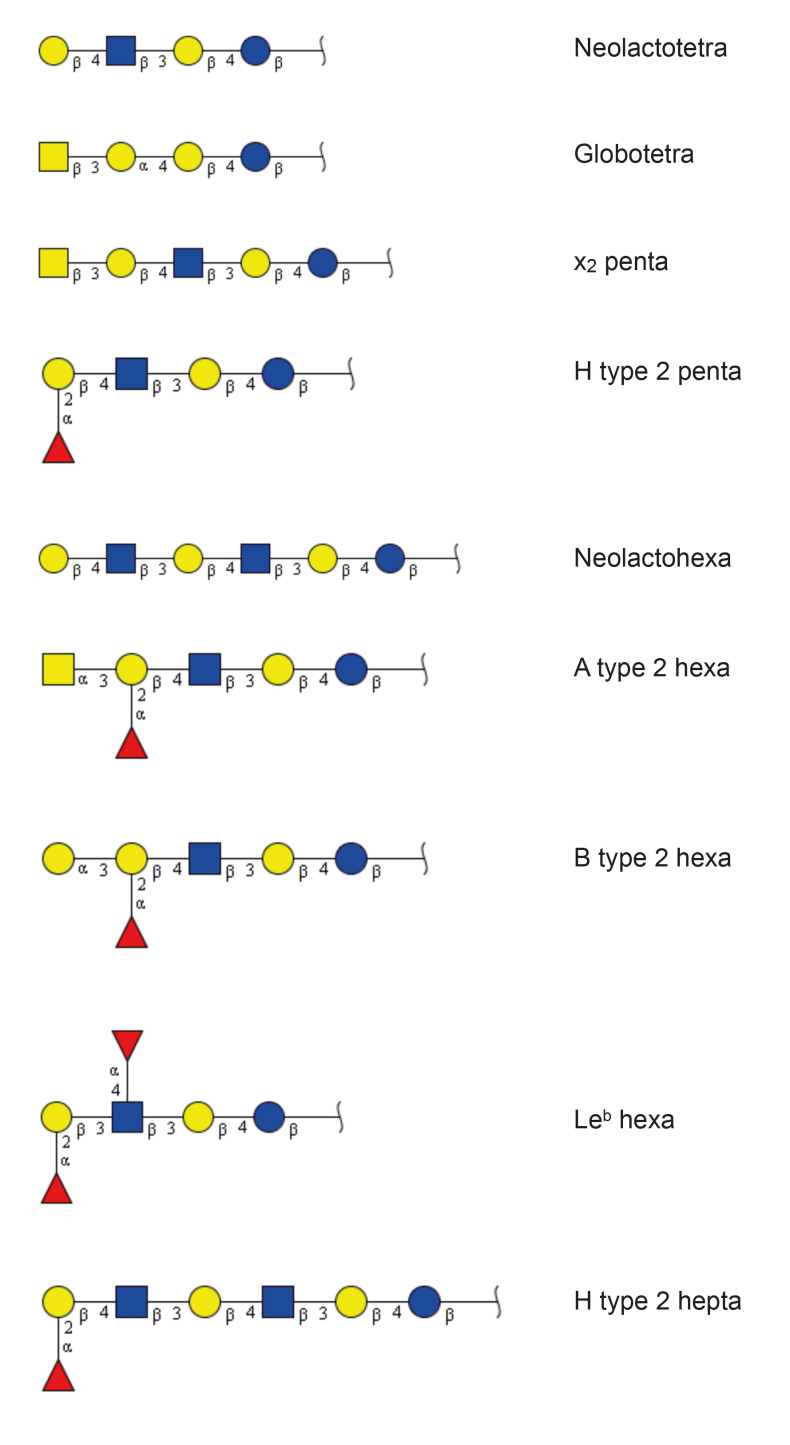
Summary of oligosaccharides derived from the non-acid glycosphingolipids from human thyroid and parathyroid glands. The proposed structures are depicted using the Symbol Nomenclature for Glycomics (SNFG) [12,13].

The oligosaccharides identified in the non-acid glycosphingolipid fractions from the human thyroid and parathyroid glands are summarized in Figure 6 and Table 2.

#### 2.2.2. Chromatogram Binding Assays

To substantiate the data from mass spectrometry, the binding of antibodies, lectins, and bacteria to the non-acid glycosphingolipids of the parathyroid and thyroid glands was thereafter tested (Figure 1B–E). The binding of Galα4Gal-recognizing P-fimbriated *E. coli* (Figure 1B) and the Galβ4GlcNAc/Fucα2Galβ4GlcNAc-binding *E. cristagalli* lectin (Figure 1C) in the tetraosylceramide region supported the presence of globotetraosylceramide and neolactotetraosylceramide, respectively, while the binding of the lectin just below the tetraosylceramide region was in line with the presence of the H type 2 pentaosylceramide. The slow-migrating compounds recognized by the *E. cristagalli* lectin were most likely neolactohexaosylceramide and/or the H type 2 heptaosylceramide. The anti-Le^b^ antibodies bound in the hexaosylceramide region in the fraction from thyroidea (Figure 1D, lane 2) were in line with the presence of the Le^b^ hexaosylceramide in this fraction. Several compounds migrating in the hexaosylceramide region and below were recognized by the anti-A antibodies (Figure 1E), supporting the presence of the A type 2 hexaosylceramide, and indicating more complex glycosphingolipids with terminal blood group A determinants.

### 2.3. Characterization of the Acid Glycosphingolipids from Human Parathyroid and Thyroid Glands

The total acid glycosphingolipid fractions were first purified by chromatography on Iatrobeads columns. For the thyroid gland, this gave one fraction (denoted by fraction T_acid_) containing a compound migrating as sulfatide on thin-layer chromatograms and resorcinol positive compounds. In the case of the parathyroid gland, one fraction containing a compound migrating as sulfatide on thin-layer chromatograms (denoted by fraction P_acid-1_), and one fraction containing resorcinol positive compounds, i.e., gangliosides (denoted by fraction P_acid-2_), were obtained.

#### 2.3.1. Mass Spectrometry

**Figure 7:**
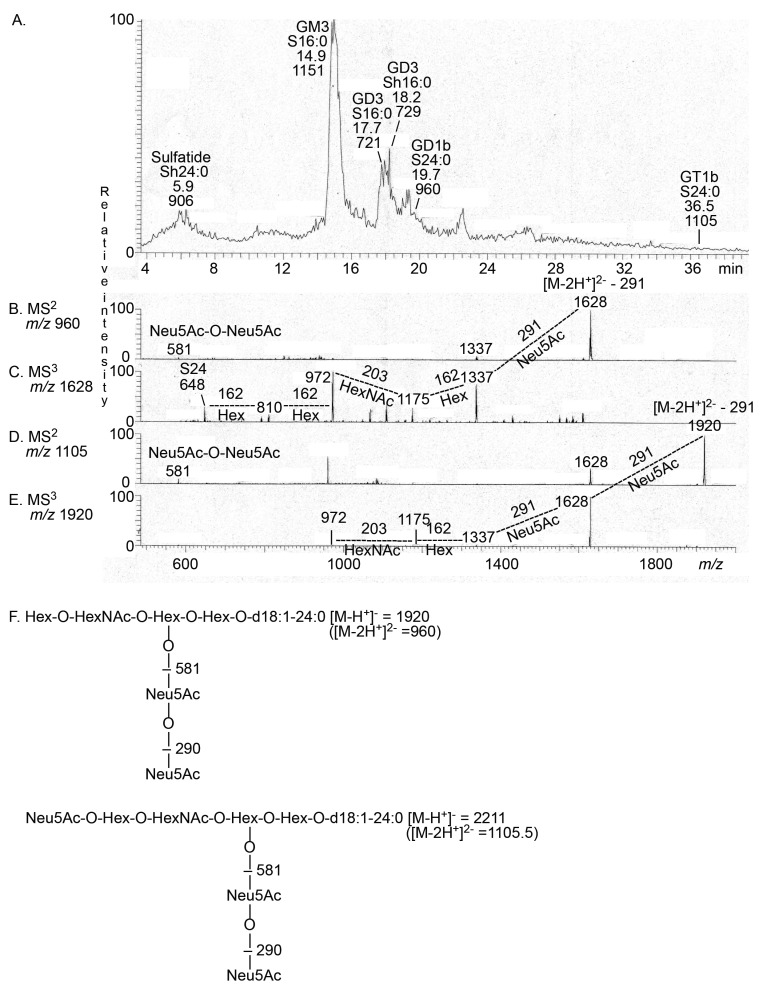
LC-ESI/MS of the total acid glycosphingolipid fraction from human thyroid gland. (**A**) Base peak chromatogram from LC-ESI/MS of the total acid glycosphingolipid fraction from human thyroid gland. (**B**) MS^2^ of the ion at *m*/*z* 960 (retention time 19.4 min). (**C**) MS^3^ of the ion at *m*/*z* 1628. (**D**) MS^2^ of the ion at *m*/*z* 1105 (retention time 36.5 min). (**E**) MS^3^ of the ion at *m*/*z* 1920. (**F**) Interpretation formula. Sulfatide, SO_3_-3Galβ1Cer; GM3, Neu5Acα3Galβ4Glcβ1Cer; GD3, Neu5Acα8Neu5Acα3Galβ4Glcβ1Cer; GD1b, Galβ3GalNAcβ4(Neu5Acα8Neu5Acα3)Galβ4Glcβ1Cer; GT1b, Neu5Acα3Galβ3GalNAcβ4(Neu5Acα8Neu5Acα3)Galβ4Glcβ1Cer. In the shorthand nomenclature for fatty acids and bases, the number before the colon refers to the carbon chain length and the number after the colon gives the total number of double bonds in the molecule. Fatty acids with a 2-hydroxy group are denoted by the prefix h before the abbreviation, e.g., h16:0. For the long chain bases, S designates sphingosine (d18:1; 1,3-dihydroxy-2-aminooctadecene) and P phytosphingosine (t18:0; 1,3,4-trihydroxy-2-aminooctadecane).

The base peak chromatogram, obtained by LC-ESI/MS, of the total acid glycosphingolipid fraction (fraction T_acid_) from the human thyroid gland (Figure 7A) had a molecular ion at *m*/*z* 906, indicating sulfatide with d18:1/h24:0 ceramide. MS^2^ of this molecular ion gave a B_1_ ion at *m*/*z* 241 and a C_1_ ion at *m*/*z* 259, confirming a SO_3_-Hex terminal (data not shown). The MS^2^ spectrum also had ions at *m*/*z* 540 and *m*/*z* 522, which are due to a loss of the fatty acyl from the molecular ion [14].

The major molecular ion in the base peak chromatogram was seen at *m*/*z* 1151. MS^2^ of this ion gave a series of Y ions (Y_0_ at *m*/*z* 536, Y_1_ at *m*/*z* 698, and Y_2_ at *m*/*z* 860), demonstrating a glycosphingolipid with Neu5Ac-Hex-Hex carbohydrate sequence and d18:1/16:0 ceramide, i.e., the Neu5Ac-GM3 ganglioside (data not shown). There were also two doubly charged molecular ions at *m*/*z* 721 and *m*/*z* 729 (which corresponded to singly charged molecular ions at *m*/*z* 1442 and *m*/*z* 1458). Here, MS^2^ identified the Neu5Ac-GD3 ganglioside with d18:1/16:0 ceramide and d18:1/h16:0 ceramide, respectively (data not shown).

The MS^2^ spectrum obtained of the doubly charged molecular ion at *m*/*z* 960 (which corresponded to a singly charged molecular ion at *m*/*z* 1920) had an ion at *m*/*z* 581, demonstrating a Neu5Ac-Neu5Ac sequence (Figure 7B). The ion series from MS^3^ of *m*/*z* 1628 (obtained by the loss of Neu5Ac from *m*/*z* 1920) identified a Hex-HexNAc-Hex-Hex sequence and d18:1/24:0 ceramide (Figure 7C). These data thus indicated a GD1b ganglioside with d18:1/24:0 ceramide.

The doubly charged molecular ion at *m*/*z* 1105.5 (which corresponded to a singly charged molecular ion at *m*/*z* 2211) indicated a ganglioside with three Neu5Ac, three Hex, one HexNAc, and d18:1/24:0 ceramide. Here, MS^2^ gave an ion at *m*/*z*, demonstrating a Neu5Ac-Neu5Ac sequence (Figure 7D), while MS^3^ of *m*/*z* 1920 (obtained by loss of Neu5Ac from *m*/*z* 2211) identified a terminal Hex-HexNAc sequence (Figure 6E). Taken together, these data suggested a GT1b ganglioside with d18:1/24:0 ceramide.

The base peak chromatogram from LC-ESI/MS of fraction P_acid-1_ from human parathyroid gland had a molecular ion at *m*/*z* 778, indicating sulfatide with d18:1/16:0 (data not shown). MS^2^ of this ion identified sulfatide (SO_3_-3Galβ1Cer) with d18:1/16:0 ceramide. This interpretation was based on the presence of a B_1_ ion at *m*/*z* 241, and a C_1_ ion at *m*/*z* 259, demonstrating a terminal SO_3_-Hex. Characteristic ions at *m*/*z* 540 and *m*/*z* 522, obtained by loss of the fatty acyl from the molecular ion, were also present [14].

**Figure 8:**
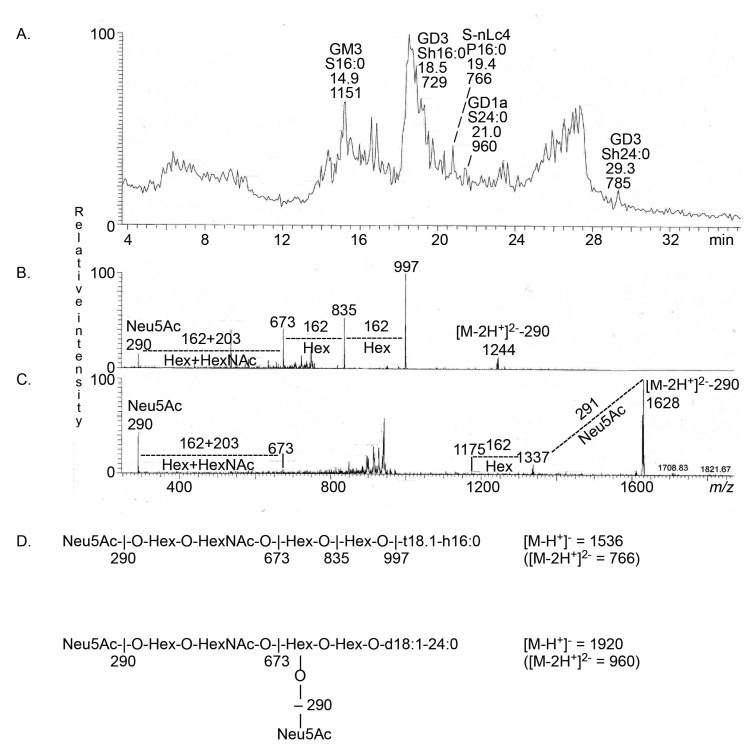
LC-ESI/MS of the acid glycosphingolipid fraction P_acid-2_ from human parathyroid gland. (**A**) Base peak chromatogram from LC-ESI/MS of the acid glycosphingolipid fraction P_acid-2_ from human parathyroid gland. (**B**) MS^2^ of the ion at *m*/*z* 766 (retention time 19.4 min). (**C**) MS^2^ of the ion at *m*/*z* 960 (retention time 21.0 min). (**D**) Interpretation formulas. GM3, Neu5Acα3Galβ4Glcβ1Cer; GD3, Neu5Acα8Neu5Acα3Galβ4Glcβ1Cer; S-nLc4, Neu5Acα3Galβ4GlcNAcβ3Galβ4Glcβ1Cer; GD1a, Neu5Acα3Galβ3GalNAcβ4(Neu5Acα3)Galβ4Glcβ1Cer. In the shorthand nomenclature for fatty acids and bases, the number before the colon refers to the carbon chain length and the number after the colon gives the total number of double bonds in the molecule. Fatty acids with a 2-hydroxy group are denoted by the prefix h before the abbreviation, e.g., h16:0. For the long chain bases, S designates sphingosine (d18:1; 1,3-dihydroxy-2-aminooctadecene) and P phytosphingosine (t18:0; 1,3,4-trihydroxy-2-aminooctadecane).

The major ion in the base peak chromatogram from LC-ESI/MS of fraction P_acid-2_ from the human parathyroid gland (Figure 8A) was a doubly charged molecular ion at *m*/*z* 729 (which corresponded to singly charged molecular ions at *m*/*z* 1458). MS^2^ of this ion identified the Neu5Ac-GD3 ganglioside with d18:1/h16:0 ceramide. MS^2^ of the doubly charged molecular ion at *m*/*z* 785 (which corresponded to singly charged molecular ions at *m*/*z* 1570) identified the Neu5Ac-GD3 ganglioside with d18:1/h24:0 ceramide. There was also a singly charged molecular ion *m*/*z* 1151. MS^2^ of this ion identified the Neu5Ac-GM3 ganglioside, as above (data not shown).

MS^2^ of the doubly charged molecular ion at *m*/*z* 766 (which corresponded to singly charged molecular ions at *m*/*z* 1532) gave an ion series at *m*/*z* 673, *m*/*z* 835, and *m*/*z* 997, demonstrating a Neu5Ac-Hex-HexNAc-Hex-Hex sequence (Figure 8B). There was also an ion at *m*/*z* 1244, which was obtained by the loss of Neu5Ac from *m*/*z* 1532. Taken together, this indicated the presence of Neu5Ac-neolactotetraosylceramide with t18:0/16:0 ceramide.

The MS^2^ spectrum obtained of the doubly charged molecular ion at *m*/*z* 960 (which corresponded to a singly charged molecular ion at *m*/*z* 1920) is shown in Figure 8C. The molecular ion at *m*/*z* 1920 again corresponded to a ganglioside with two Neu5Ac, three Hex, one HexNAc, and d18:1/24:0 ceramide. Here, the ion at *m*/*z* 673 demonstrated a terminal Neu5Ac-Hex-HexNAc sequence. No ion at *m*/*z* 581 demonstrating a Neu5Ac-Neu5Ac sequence was found. Thus, the GD1a ganglioside with d18:1/24:0 ceramide was identified.

**Figure 9:**
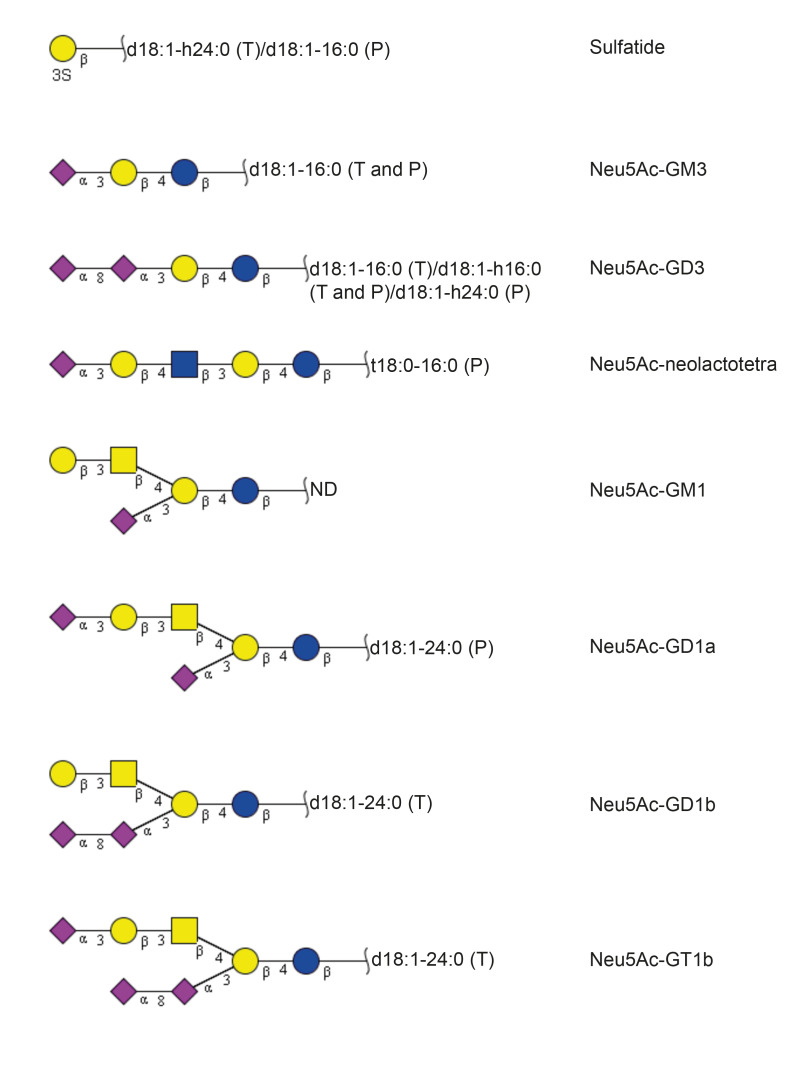
Summary of the acid glycosphingolipids from human thyroid and parathyroid glands. The proposed structures are depicted using the Symbol Nomenclature for Glycomics (SNFG) [12,13]. In the shorthand nomenclature for fatty acids and bases, the number before the colon refers to the carbon chain length and the number after the colon gives the total number of double bonds in the molecule. Fatty acids with a 2-hydroxy group are denoted by the prefix h before the abbreviation, e.g., h16:0. For the long chain bases, d18:1 is sphingosine (1,3-dihydroxy-2-aminooctadecene) and t18:0 is phytosphingosine (1,3,4-trihydroxy-2-aminooctadecane). T denotes thyroid gland, PT denotes parathyroid gland, and ND denotes not determined.

The glycosphingolipids characterized in the acid fractions from the human thyroid and parathyroid glands are summarized in Table 3 and Figure 9.

#### 2.3.2. Chromatogram Binding Assays

The binding of cholera toxin B-subunits and monoclonal antibodies to the acid fractions isolated from human thyroid and parathyroid glands is shown in Figure 2, lanes 1 and 2. Here, the cholera toxin B-subunits bound to both fractions (Figure 2B), indicating the presence of the GM1 ganglioside, although this ganglioside was not characterized by mass spectrometry. A distinct binding of anti-GD3 monoclonal antibodies to both fractions was also obtained (Figure 2C). The anti-GD1a monoclonal antibodies bound only to the acid glycosphingolipids from the parathyroid gland (Figure 2D, lane 2), in line with the results from mass spectrometry. Finally, binding of the monoclonal antibodies directed against Neu5Acα3-neolacto and Neu5Acα6-neolacto sequences to the acid fractions isolated from both human thyroid and parathyroid glands was obtained (Figure 2E,F).

### 2.4. Immunohistochemistry

**Figure 10:**
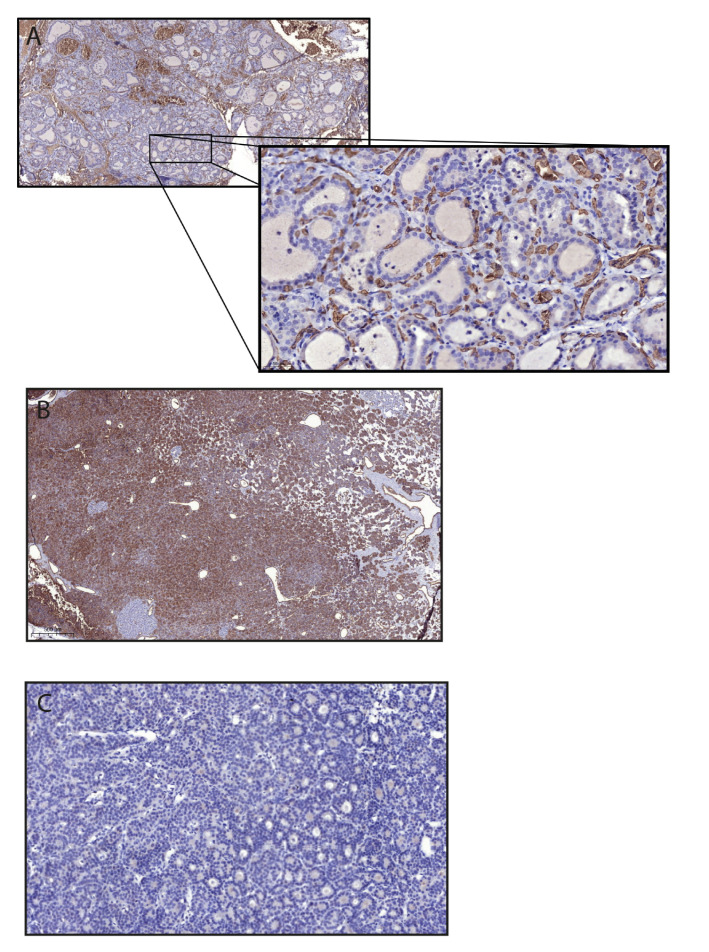
Immunohistochemical evaluation of blood group antigen expression in thyroid (**A**) and parathyroid (**B**,**C**) glands collected from patients with blood group A (**A**,**B**) and O (**C**). (**A**) Illustrates positive anti-blood group A antibody staining in the supportive and vascular tissue of the thyroid gland. The magnification demonstrates the expression of blood group A antigen in the c-cells. No expression of blood group A antigens was found in the follicular cells. (**B**) Illustrates the high expression of blood group A antigens in the parathyroid cells from a patient with blood group A. (**C**) Shows weak cytoplasmatic expression of H type 1 in a blood group O individual.

Immunohistochemical evaluation showed a positive anti-blood group A staining of the thyroid and parathyroid glands (Figure 10A,B), consistent with the patient’s blood group status. Blood group A antigens were present in the c-cells as well as in the supportive and vascular tissue of the thyroid glands, but not in the follicular cells (Figure 10A). A high expression of blood group A antigens was found on the cell surface and in the cytoplasm of the parathyroid cells (Figure 10B). One individual was blood group O and consequently lacked expression of blood group A and B antigens, but demonstrated weak cytoplasmatic staining with anti-H-type 1 antibodies (Figure 10C). All other samples were collected from blood group A individuals and showed no expression of blood group B or H type 1 antigens.

**Figure 11:**
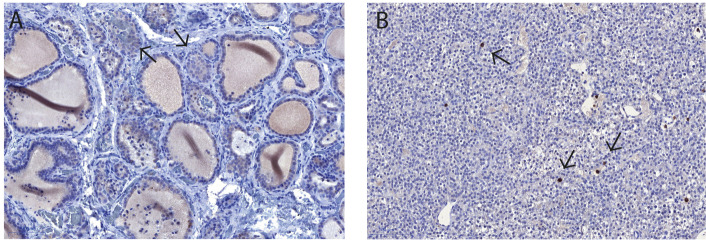
Immunohistochemical evaluation of the GD1a expression of the human thyroid (**A**) and parathyroid gland (**B**). (**A**) Weak cytoplasmatic staining was found in the follicular cells and c-cells (arrows) in two of the thyroid glands. (**B**) Demonstrates single positive cells with high expression of GD1a on the cell surface evenly distributed in the parathyroid tissue (arrows).

To further characterize the presence and distribution of gangliosides, the expression of GD1a was evaluated. Weak cytoplasmatic staining was found in two of the thyroid samples (Figure 11A), and strong staining was found on the cell surface of single cells in the parathyroid gland (Figure 11B).

## 3. Discussion

In this study acid and non-acid glycosphingolipids were isolated from human thyroid and parathyroid glands, and, with focus on complex compounds, characterized with mass spectrometry, and binding of carbohydrate-recognizing ligands (antibodies, lectins and bacteria). The glycosphingolipid patterns of human parathyroid and thyroid glands were very similar. The major acid glycosphingolipids were identified as sulfatide and the gangliosides GM3, GD3, GD1a, GD1b, GT1b, and Neu5Ac-neolactotetraosylceramide, and the major complex non-acid glycosphingolipids as globotriaosylceramide and globoside, consistent with previous studies of the thyroid gland [5,6,7,8,9]. In addition, we found neolactotetra- and neolactohexaosylceramide, the x_2_ glycosphingolipid, and complex glycosphingolipids with terminal blood group O and A determinants in both tissues. Glycosphingolipids with blood group Le^b^ determinant were also identified in the thyroid gland, and, in the parathyroid sample, a glycosphingolipid with terminal blood group B determinant was characterized.

The immunohistochemical analysis verified the expression of blood group A antigens in thyroid glands, mainly present in the supportive and vascular tissue, but also found in the c-cells. Previous immunohistochemical studies have reported that there are no blood group ABO determinants in normal thyroids [15,16]. The reason for this discrepancy is unclear, but might depend on the monoclonal antibodies used. Alternatively, this is due to changes in the ABO antigens, caused by differentiation and/or maturation, although altered glycosylation is usually associated with malignant transformation [17]. Extensive expression of blood group A was seen in the parathyroid cells, in analogue with previous studies [18,19].

In the acid fractions, the GD1b ganglioside was identified in the thyroid tissue by mass spectrometry, whereas the GD1a ganglioside was characterized in the parathyroid tissue. A strong expression of the GD1a ganglioside on the cell surface of single cells in the parathyroid gland was found by immunohistochemistry, while a weak cytoplasmatic expression was present in the thyroid samples. Potentially, GD1a could be a marker for parathyroid cells that can be used for diagnostics and therapeutic purposes. This should be further evaluated.

Interestingly, a specific association of the both human and porcine thyrotropin/TSH receptor with a ganglioside has been reported [20,21,22], and, in the Fisher rat thyroid cell line, this ganglioside was subsequently identified as lactonized Neu5Acα3Galβ3GalNAcβ4(Neu5Acα3)Galβ4Glcβ1Cer (GD1a-lactone). However, no up-to-date information about this issue is available.

Furthermore, the thyrotropin/TSH receptor has been found to reside in GM1 ganglioside-enriched lipid rafts in Chinese hamster ovary cells [23]. Subsequently, it was demonstrated that, for TSH receptor-transfected cells and rat thyrocytes, a significant proportion of thyrotropin/TSH receptors reside within lipid microdomains, and that multimerization of thyrotropin/TSH receptors is regulated within the lipid rafts [24]. In addition, the negative effects of the insecticide dichlorodiphenyltrichloroethane (DDT) on TSH receptor activation are due to the depletion of these rafts’ phospholipid and cholesterol contents, which prevents the internalization of the TSH receptor [25].

In summary, this study presents, for the first time, a thorough characterization of the glycosphingolipid composition of the human parathyroid and thyroid glands. We show that the glycosphingolipid patterns of the parathyroid and thyroid glands are more complex than previously described. Furthermore, glycosphingolipids carrying ABO blood group antigens are present in both glands. Our findings provide a platform for further studies of alterations of cell surface glycosphingolipids in thyroid and parathyroid cancers.

## 4. Materials and Methods

### 4.1. Glycosphingolipid Preparations

Tissue samples from nine patients with diseased parathyroid glands, and thyroid tissue from one patient with Grave’s disease, were collected with approval from the Regional Ethics Committee of Gothenburg (Dnr 1103-17), Uppsala Local Ethical Committee (Dnr 2015-180), and Swedish Ethical Review Authority (No. 2020-06142), following the Declaration of Helsinki and the General Data Protection Regulation (GDPR). All patients were given written and verbal information before signing informed consent to participate and agreeing to the use of the information in research.

The parathyroid glands were from four blood group O individuals, two blood group A individuals, two blood group B individuals, and one blood group AB individual. The thyroid gland was from a blood group A individual.

The parathyroid glands were pooled and lyophilized, giving a dry weight of 4.6 g. The dry weight of the thyroid tissue was 5.2 g after lyophilization. The isolation of total acid and total non-acid glycosphingolipids was done by the method described by Karlsson [26]. The lyophilized materials were extracted in two steps in a Soxhlet apparatus with chloroform and methanol (in proportions 2:1 and 1:9, by volume). The extracts were subjected to mild alkaline hydrolysis and dialysis, followed by separation on a silicic acid column. Acid and non-acid glycosphingolipid fractions were obtained by chromatography on a DEAE-cellulose column. In order to separate the non-acid glycosphingolipids from alkali-stable phospholipids, the non-acid fractions were acetylated and separated on a second silicic acid column, followed by deacetylation and dialysis. Final purifications were done by chromatographies on DEAE-cellulose and silicic acid columns.

The amounts of total acid and non-acid glycosphingolipids obtained from the parathyroid and thyroid glands are given in Table 1.

The total glycosphingolipid fractions were characterized by liquid chromatography-electrospray mass spectrometry (LC-ESI/MS), thin-layer chromatography, and binding of carbohydrate-recognizing ligands (antibodies, lectins, and bacteria) in chromatogram-binding assays (see below).

The total acid glycosphingolipid fractions were thereafter purified by chromatography on Iatrobeads (Iatron Laboratories, Tokyo, Japan) columns eluted with increasing amounts of methanol in chloroform. This gave, in the case of the parathyroid gland, one fraction containing a compound migrating as sulfatide on thin-layer chromatograms (denoted by fraction P_acid-1_), and one fraction containing resorcinol positive compounds, i.e., gangliosides (denoted by fraction P_acid-2_). In the case of the thyroid gland, one fraction containing both a compound migrating as sulfatide on thin-layer chromatograms and resorcinol positive compounds was obtained. This fraction was denoted by T_acid_.

### 4.2. Reference Glycosphingolipids

Total acid and non-acid glycosphingolipid fractions were isolated as described [26]. Individual glycosphingolipids were isolated by repeated chromatography on silicic acid columns and by high-pressure liquid chromatography (HPLC), and identified by mass spectrometry [10,27] and ^1^H-NMR spectroscopy [28].

### 4.3. Thin-Layer Chromatography

Thin-layer chromatography was done on aluminum- or glass-backed silica gel 60 high performance thin-layer chromatography plates (Merck, Darmstadt, Germany). Glycosphingolipid mixtures (40 μg) or pure glycosphingolipids (4 μg) were applied to the plates and eluted with chloroform, methanol, and water (in proportions 60:35:8, by volume). Chemical detection was done with anisaldehyde [29].

### 4.4. Chromatogram Binding Assays

The mouse monoclonal antibodies, and other carbohydrate-recognizing ligands, tested for binding to the glycosphingolipids of the human parathyroid and thyroid glands in the chromatogram-binding assay are given in Table 4. Binding of the monoclonal antibodies to glycosphingolipids separated on thin-layer chromatograms was done as described [30,31]. Chromatograms with separated glycosphingolipids were dipped for 1 min in diethylether and *n*-hexane (in proportion 1:5, by volume) containing 0.5% (*w*/*v*) polyisobutylmethacrylate (Sigma-Aldrich, St. Louis, MO, USA). After drying, the chromatograms were soaked in phosphate-buffered saline (PBS), pH 7.3, containing 2% bovine serum albumin and 0.1% NaN_3_ (Solution A), for 2 h at room temperature. Suspensions of monoclonal antibodies diluted in Sol. A (the dilution used for each monoclonal antibody is given in Table 4) were sprinkled over the chromatograms, followed by incubation for 2 h at room temperature. Then, they were washed with PBS, followed by a second 2 h incubation with ^125^I-labeled (labeled by the Iodogen method according to the instruction of the manufacturer (Pierce/Thermo Fischer Scientific, Stockholm, Sweden)) rabbit anti-mouse antibodies (DakoCytomation Norden A/S, Glostrup, Denmark), diluted to 2 × 10^6^ cpm/mL in Sol. A. Finally, the plates were washed six times with PBS. Dried chromatograms were autoradiographed for 12–24 h using XAR-5 X-ray films (Carestream/Sigma-Aldrich, St. Louis, MO, USA).

Binding of ^125^I-labeled *Erythrina cristagalli* lectin (Sigma-Aldrich, St. Louis, MO, USA) and cholera toxin B-subunits (List Labs., Campbell, CA, USA), and ^35^S-labeled P-fimbriated *Escherichia coli* strain 291-15, to glycosphingolipids on thin-layer chromatograms were done as described [34,35,36].

### 4.5. Endoglycoceramidase Digestion and LC-ESI/MS

Endoglycoceramidase II from *Rhodococcus* spp. (Takara Bio Europe S.A., Gennevilliers, France) was used for hydrolysis of the non-acid glycosphingolipids. The glycosphingolipids (50 μg) were resuspended in 100 μL 0.05 M sodium acetate buffer, pH 5.0, containing 120 μg sodium cholate, and sonicated briefly. Thereafter, 1 mU of enzyme was added, and the mixture was incubated at 37 °C for 48 h. The reaction was stopped by addition of chloroform, methanol, and water, in the final proportions 8:4:3 (by volume). The oligosaccharide-containing upper phase thus obtained was separated from detergent on a Sep-Pak QMA cartridge (Waters, Milford, MA, USA). The eluant containing the oligosaccharides was dried under nitrogen and under vacuum.

Part of the oligosaccharide samples were reduced by adding 20 μL of 200 mM NaBH_4_ in 50 mM KOH to the samples and incubating at 50 °C for 2 h [10]. The samples were then acidified by adding 10 μL of glacial acetic acid, and the oligosaccharides were desalted by cation exchange chromatography, and thereafter evaporated to dryness.

The glycosphingolipid-derived oligosaccharides were resuspended in 50 μL of water and analyzed by LC-ESI/MS, as described in [10]. The oligosaccharides were separated on a column (100 × 0.250 mm) packed in-house with 5 μm porous graphite particles (Hypercarb, Thermo-Hypersil, Runcorn, UK). An autosampler, HTC-PAL (CTC Analytics AG, Zwingen, Switzerland) equipped with a cheminert valve (0.25 mm bore) and a 2 μL loop, was used for sample injection. An Agilent 1100 binary pump (Agilent Technologies, Palo Alto, CA, USA) delivered a flow of 250 μL/min, which was split down in an 1/16” microvolume-T (0.15 mm bore) (Vici AG International, Schenkon, Switzerland) by a 50 cm × 50 μm i.d. fused silica capillary before the injector of the autosampler, allowing approximately 3–5 μL/min through the column. The oligosaccharides (3 μL) were injected onto the column and eluted with an acetonitrile gradient (A—10 mM ammonium bicarbonate; B—10 mM ammonium bicarbonate in 80% acetonitrile). The gradient (0–45% B) was eluted for 46 min, followed by a wash step with 100% B, and equilibration of the column for 24 min. A 30 cm × 50 μm i.d. fused silica capillary was used as transfer line to the ion source.

The oligosaccharides were analyzed in negative ion mode on an LTQ linear quadrupole ion trap mass spectrometer (Thermo Electron, San José, CA, USA). The IonMax standard ESI source on the LTQ mass spectrometer was equipped with a stainless steel needle kept at –3.5 kV. Compressed air was used as nebulizer gas. The heated capillary was kept at 270 °C, and the capillary voltage was –50 kV. A full scan (*m*/*z* 380–2 000, 2 microscans, maximum 100 ms, target value of 30,000) was performed, followed by data dependent MS^2^ scans of the three most abundant ions in each scan (2 microscans, maximum 100 ms, target value of 10,000). The threshold for MS^2^ was set to 500 counts. Normalized collision energy was 35%, and an isolation window of 3 u, an activation q = 0.25, and an activation time of 30 ms, was used. Selected fractions were also analyzed at *m*/*z* 1300–2000. Data acquisition and processing were conducted with Xcalibur software (Thermo Scientific, Waltham, MA, USA; Version 2.0.7). Raw data were uploaded on (https://glycopost.glycosmos.org/entry/GPST000184) accessed date 10 May 2021.

Manual assignment of glycan sequences was done on the basis of knowledge of mammalian biosynthetic pathways with the assistance of the Glycoworkbench tool (Version 2.1), and by comparison of retention times and MS^2^ spectra of oligosaccharides from reference glycosphingolipids [10].

### 4.6. LC-ESI/MS of Native Acid Glycosphingolipids

The native acid glycosphingolipid fractions were analyzed by LC-ESI/MS as described [37]. Aliquots of the glycosphingolipid fractions were dissolved in methanol and acetonitrile in proportion 75:25 (by volume) and separated on a 200 × 0.250 mm column, packed in-house with 5 μm polyamine II particles (YMC Europe GmbH, Dinslaken, Germany). An autosampler, HTC-PAL (CTC Analytics AG, Zwingen, Switzerland) equipped with a cheminert valve (0.25 mm bore) and a 2 µL loop, was used for sample injection. An Agilent 1100 binary pump (Agilent technologies, Palo Alto, CA, USA) delivered a flow of 250 µL/min, which was split down in a 1/16” microvolume-T (0.15 mm bore) (Vici AG International, Schenkon, Switzerland) by a 50 cm × 50 µm i.d. fused silica capillary before the injector of the autosampler, allowing approximately 2–3 µL/min through the column. Samples were eluted with an aqueous gradient (A: 100% acetonitrile to B: 10 mM ammonium bicarbonate). The gradient (0–50% B) was eluted for 40 min, followed by a wash step with 100% B, and equilibration of the column for 20 min. The samples were analyzed in negative ion mode on a LTQ linear quadropole ion trap mass spectrometer (Thermo Electron, San José, CA), with an IonMax standard ESI source equipped with a stainless steel needle kept at –3.5 kV. Compressed air was used as nebulizer gas. The heated capillary was kept at 270 °C, and the capillary voltage was –50 kV. A full scan (*m*/*z* 500–1800, two microscan, maximum 100 ms, target value of 30,000) was performed, followed by data-dependent MS^2^ scans (two microscans, maximun 100 ms, target value of 10.000) with a normalized collision energy of 35%, isolation window of 2.5 units, activation q= 0.25, and activation time 30 ms. The threshold for MS^2^ was set to 500 counts. Data acquisition and processing were conducted with Xcalibur software (Thermo Scientific, Waltham, MA, USA; Version 2.0.7). Raw data were uploaded on (https://glycopost.glycomos.org/entry/GPST000184) accessed date 10 May 2021.

Data acquisition and processing were conducted with Xcalibur software (Version 2.0.7). Manual assignment of glycosphingolipid sequences was done with the assistance of the Glycoworkbench tool (Version 2.1), and by comparison of retention times and MS^2^ spectra of reference glycosphingolipids.

### 4.7. Immunohistochemistry

Samples from human parathyroid glands (n = 3), and thyroid glands (n = 3) were fixed in buffered 4% paraformaldehyde, dehydrated, and embedded in paraffin. Subsequently, 4 µm sections were mounted on Superfrost Plus glass slides (Menzel) and microwave treated for antigen retrieval. Immunostaining was performed after pretreatment with Diva Decloaker 20X (Biocare Medical, Pacheco, CA, USA) at 95 °C for 40 min and blocking reagent Peroxidazed 1 (Biocare Medical). The primary antibodies used were anti-blood group A antigen (clone HE-193, dilution 1:50, cat. No. ab2521, Abcam, Cambridge, England), anti-blood group B antigen (HEB-29, 1:50, ab2524, Abcam), anti-H-type-1 (17–206, 1:50, ab3355, Abcam), and anti-GD1a ganglioside (GD1a-1, 1:50, MAB5606Z, Sigma-Aldrich, St. Louis, MO, USA). MACH 1 Universal HRP-polymer kit (Biocare Medical), including Betazoid DAB substrate and blocking reagent Background Sniper, was used for detection of bound antibodies. Nuclei were counterstained with Tacha’s automated hematoxylin (Biocare Medical).

## Figures and Tables

**Table 1:** Glycosphingolipid preparations.

	Dry Weight	Total Acid Glycosphingolipids	Total Non-Acid Glycosphingolipids	mg Acid Glycosphingolipids/g Dry Weight	mg Non-Acid Glycosphingolipids/g Dry Weight
Parathyroid gland	4.6 g	5.0 mg	19.1 mg	1.1	4.1
Thyroid gland	5.2 g	10.2 mg	12.8 mg	2.0	2.5

**Table 2:** Glycosphingolipid-derived oligosaccharides from the total non-acid fractions identified by LC-ESI/MS.

Trivial Name	Structure	Parathyroid Gland	Thyroid Gland
Neolactotetra (nLc4)	Galβ4GlcNAcβ3Galβ4Glc	+	+
Globotetra (Gb4)	GalNAcβ3Galα4Galβ4Glc	+	+
x_2_ penta (x_2_)	GalNAcβ3Galβ4GlcNAcβ3Galβ4Glc	+	+
H type 2 penta (H5-2)	Fucα2Galβ4GlcNAcβ3Galβ4Glc	+	+
Neolactohexa (nLc6)	Galβ4GlcNAcβ3Galβ4GlcNAcβ3Galβ4Glc	+	+
A type 2 hexa (A6-2)	GalNAcα3(Fucα2)Galβ4GlcNAcβ3Galβ4Glc	+	+
B type 2 hexa (B6-2)	Galα3(Fucα2)Galβ4GlcNAcβ3Galβ4Glc	+	-
Le^b^ hexa (Le^b^-6)	Fucα2Galβ3(Fucα4)GlcNAcβ3Galβ4Glc	-	+
H type 2 hepta (H7-2)	Fucα2Galβ4GlcNAcβ3Galβ4GlcNAcβ3Galβ4Glc	+	+

**Table 3:** Glycosphingolipids in the total acid fractions identified by LC-ESI/MS.

Trivial Name	Structure	Parathyroid Gland	Thyroid Gland
Sulfatide	SO_3_-3Galβ1Cer	+	+
Neu5Ac-GM3	Neu5Acα3Galβ4Glcβ1Cer	+	+
Neu5Ac-GD3	Neu5Acα8Neu5Acα3Galβ4Glcβ1Cer	+	+
Neu5Ac-nLc4	Neu5Acα3Galβ4GlcNAcβ3Galβ4Glcβ1Cer	+	+
Neu5Ac-GM1	Galβ3GalNAcβ4(Neu5Acα3)Galβ4Glcβ1Cer	+	+
Neu5Ac-GD1a	Neu5Acα3Galβ3GalNAcβ4(Neu5Acα3)Galβ4Glcβ1Cer	+	-
Neu5Ac-GD1b	Galβ3GalNAcβ4(Neu5Acα8Neu5Acα3)Galβ4Glcβ1Cer	-	+
Neu5Ac-GT1b	Neu5Acα3Galβ3GalNAcβ4(Neu5Acα8Neu5Acα3)Galβ4Glcβ1Cer	-	+

**Table 4:** Carbohydrate binding ligands used in chromatogram binding assays.

Ligand	Clone	Manufacturer	Dilution	Binding Specificity
Cholera toxin B-subunits	-	List Labs	-	Galβ3GalNAcβ4(Neu5Acα3)Galβ4Glc
Anti-GD3	MB3.6	BD Biosciences	1:100	Neu5Acα8Neu5Acα3Galβ4Glc
Anti-GD1a	GD1a-1	Sigma-Aldrich	1:100	Neu5Acα3Galβ3GalNAcβ4(Neu5Acα3)Galβ4Glc
Anti-Neu5Acα3-nL4	LM1:1a	[32]	1:1000	Neu5Acα3Galβ4GlcNAc
Anti-Neu5Acα6-nL4	LM4:2	[33]	1:100	Neu5Acα6Galβ4GlcNAc
P-fimbriated *E. coli*	-	[34]	-	Galα4Gal
*E. cristagalli* lectin		Sigma-Aldrich	-	Galβ4GlcNAc/Fucα2Galβ4GlcNAc
Anti-Lewis ^b^	BG-6/T218	Signet/Covance	1:100	Fucα2Galβ3(Fucα4)GlcNAc
Anti-blood group A	HE-195	Sigma-Aldrich	1:500	GalNAcα3(Fucα2)Gal

## Data Availability

Mass spectrometry raw data has been uploaded on (https://glycopost.glycosmos.org/entry/GPST000184) accessed date 10 May 2021.

## Abbreviations

DDT, dichlorodiphenyltrichloroethane; 4D-CT, four-dimensional computed tomography; GDPR, General Data Protection Regulation; HPLC, high-pressure liquid chromatography; LC-ESI/MS, liquid chromatography electrospray ionization mass spectrometry; PBS, phosphate-buffered saline.
